# Women and Nursing

**Published:** 1920-07-03

**Authors:** 


### Women and Nursing.

### Are Conditions Tyrannous and Antiquated?

THE shortage of probationers for the nursing profession is exercising many minds, and has been attributed to various causes. It is well to have an expression of opinion from women on what is so largely a woman's profession, and for that reason serious attention should be given to an article which recently appeared in the *Woman's Leader*. We neither accept its view nor controvert it, but simply put it forward as one that cannot be ignored by the responsible authorities, for it affects the well-being of the community very closely. The standpoint taken is this: nursing is not now, as it used to be, one of few callings open to women. Women are not deterred by fear of the hard work involved, but they look to professions where there is greater freedom. It is strongly doubted whether there is need for such rigidly, inelastic discipline for the training of a woman's nursing talent as is enforced, and though discipline is necessary, petty tyranny and trifling rules and restrictions for off-duty times are intolerable, placing probationers on an intellectual level with children. Such methods are out of date to-day. A probationer is asked to sign an entirely one-sided agreement after she has completed her trial. She must promise to serve the hospital for three or four years; she can be dismissed without notice on most elastic terms, and there have been many cases where it has taken the authorities two years to find that a probationer is not suitable.

Objection is taken to the rigid separation of doctors and nurses, which does not apply to doctors and women students, though nurses and students are frequently of exactly the same social standing The general plea is that fairer, more enlightened, freer, and more up-to-date methods must be employed if the modern young woman is to be attracted to one of the finest forms of social service. A protest is entered against the theory of a training for nursing which "either makes or breaks a woman." Students of every other art are universally acknowledged to be entitled to youth's privilege of enjoying youth, and the time has come when the authorities should at least try the experiment of granting the same privilege to student nurses. That is the case as presented by a paper representing the women's point of view, and it is not negligible.

